# Supplementary material for: Personality types revisited–a literature-informed and data-driven approach to an integration of prototypical and dimensional constructs of personality description
Source: PLoS One. 2021 Jan 7;16(1):e0244849. doi: 10.1371/journal.pone.0244849 (PMC7790254; doi:10.1371/journal.pone.0244849)
Supplement: Attachment — Submitted filename: Response to the Reviewers.docx [file pone.0244849.s001.docx]

In the following, all line numbers refer to the version of the manuscript with tracked changes.

Reviewer 1:

1) For example, the abstract only refers to “a large representative German dataset” without providing the N? What is the abbreviation Ward/k standing for? In general, I encourage the authors to rephrase the abstract and parts of the introduction as a service for the reader.

*Response: We rephrased the abstract so that it sounds less technical. It was a very valuable	suggestion to reread and rephrase both abstract and introduction from the readers perspective. We must admit that there were several not really reader-friendly technical parts not only in the introduction, we therefore streamlined the whole manuscript with respect to this problem.*

2) General comment: During the introduction on the first 3-4 pages, I feel somewhat lost as reader. I suppose the authors could sharpen up this part. It may also be helpful to link the rationale to some more familiar/contemporary theory and research within the field. The 10 first pages seem to work as a review of the literature. The aim is not presented until page 9 line 207.

*Response: Indeed, the purpose of the literature review became not clear in the introduction, so reviewer 2 even suggested to delete it completely. But the literature review is actually a very central part, as it has the purpose to (1) identify personality prototypes that were consistently found in previous literature to calculate averaged prototypical profiles based on these previous findings, (2) link the prototypes found in our study to previous findings and therefore (3) verify construct validity of our findings. We now added a paragraph in ll. 81-92 explicitly stating the aim of the study and the purpose of the literature review, structured the introduction in three parts (general, review part, research gap and novelty part) to guide the reader through this part of the study and added a recent study based on HEXACO data and agency-communion concepts.*

3) It could be helpful with a more clear distinguishing between types and traits?

*Response: We now rephrased the first sentences explaining the two approaches and hope that they are now clearer and distinguish the two approaches better.*

4) Page 6, line 127: “In a recent nature human behavior publication” – are the authors referring to a journal here or a particular issue or paper? It is not evident.

5) Page 7: ”total N of 1560418” – please use comma separators.

*Response: We rephrased this part of the Manuscript.*

6) The authors cite the HiTOP and related scientific papers (e.g., Forbush et al, Kotov et al., Krueger et al.). However, the authors did not relate their findings or discussions to the more authoritative diagnostic frameworks such as the approved ICD-11 dimensional classifications of PDs as well as the DSM-5 alternative model – with particular emphasis on their trait systems.

*Response: Thanks very much for this suggestion. We were hesitating to open up this topic in the discussion as the manuscript was already containing a lot of references to different methodological, psychological and clinical research fields. But your comment encouraged us to include a new paragraph (ll.944-950) with a reference to the AMPD and ICD-11 maladaptive trait models and also state the hypothesis that these may also align around (maladaptive) personality prototypes. We further include a study discussing person-oriented approaches in the research field of personality disorders.*

7) On page 4 the authors write: “it can be said that the human goal is to be as undercontrolled as possible and as overcontrolled as necessary. When one is more undercontrolled than is adaptively effective or more overcontrolled than is adaptively required, one is not resilient”

In relation to “resilience”, it is remarkable that the authors have not related their findings or discussion to Fonagy and Sharp as well as Caspi’s P-factor (see references below). I particularly refer to the P-factor as an index of insufficient resilience, which may be something that could be more clearly incorporated into the manuscript?

Caspi, A., Houts, R. M., Belsky, D. W., & Goldman-mellor, S. J. (2015). The p factor: One general psychopathology factor in the structure of psychiatric disorders? Clinical Psychological Science, 2(2), 119–137. https://doi.org/10.1177/2167702613497473.

Sharp, C., Wright, A. G. C., Fowler, J. C., Frueh, B. C., Allen, J. G., Oldham, J., & Clark, L. A. (2015). The structure of personality pathology: Both general (‘g’) and specific (‘s’) factors? Journal of Abnormal Psychology, 124(2), 387–398. https://doi.org/10.1037/abn0000033

Fonagy, P., Luyten, P., Allison, E., & Campbell, C. (2017). What we have changed our minds about: Part 1. Borderline personality disorder as a limitation of resilience. Borderline Personality Disorder and Emotion Dysregulation, 4(1), 11. <https://doi.org/10.1186/s40479-017-0061-9>

*Response: Again, thanks very much for this suggestion. We were also hesitating to include more literature from the personality structure/functioning research field. We now have rephrased the whole paragraph on the resilient personality type in ll. 889 – 905 and included both references to Fonagy and Kernberg as well as the AMPD Criterion A construction paper by Morey et al. (2011). We also include the Caspi-Paper on the p-factor. Although impairments in personality functioning are strongly correlated with high comorbidity and a high p-factor, we do not think that it boils down to the same thing and therefore also include a reference to a paper discussing the difference between quality of life, psychosocial functioning and personality functioning. We think that impairments in personality functioning mainly result in poor mental health through an interplay between negative (interpersonal) life events and a low ability to cope or regulate emotions while having no access to psychosocial support or treatments (independent from wether the latter is due to within-person or external barriers).*

Reviewer 2:

General: 1. it includes a long part, not consistent with the title and the abstract, which can be eliminated; 2 the description of the statistical methodology is poorly understood by colleagues who are not experts in data analysis. The text is weighted and complex to read.

*Response: It was a very valuable suggestion to reread and rephrase both abstract and introduction from the readers perspective. We must admit that there were several not really reader-friendly technical parts not only in the introduction, we therefore restructured and streamlined both introduction and abstract and adjusted the title of the manuscript so that it conforms better with the structure and approach of the study.*

1) from row 48 to row 51: The difference between the two approaches should be clearly explained

*Response: We now have added sentences describing the differences in more detail ll. 48-61*

2) from row 69 to row 70: The Q procedure should be clearly explained

*Response: We now have added a paragraph explaining the Q-Sort procedure.*

3) from row 88 to row 91: I would delete this sentence

*Response: Yes, this sentence added some redundancy, we now have deleted it.*

4) from row 127 to row 130: I would explain this study in more detail

*Response: We now explain the Gerlach et al. (2018) study in more detail and added a further study on personality prototypes using large HEXACO datasets in ll. 167-180*

5) rom row 131 to row 133: the reasons for this choice should be explained

*Response: We now have added an explanation for this decision in ll. 183-187, which we primarily have made to ensure the quality of the included studies that we used to calculate the averaged personality profiles.*

6) from row 143 to row 206: I would eliminate this part (it seems to me, to all intents and purposes, something that may belong to an interesting review of the literature. this part proposed in this stringed way is obviously inadequate, inconsistent with the title and unnecessarily burdens the text) Clearly this implies the elimination also of figure 1 and of the results and discussion that refer to the comparison between figure 1 and figure 2

*Response: Indeed, the purpose of the literature review became not clear in the introduction in the previous version of the Manuscript. But the literature review is actually a very central part, as it has the purpose to (1) identify prototypes that were consistently found in previous literature, (2) calculate averaged prototypical profiles based on these previous findings, (3) link the prototypes found in our study to previous findings and therefore (4) verify construct validity of our findings. The procedure of averaging previously replicated personality profiles allows us to reliably link the personality prototypes found in our study to previous empirical evidence, an important analysis step lacking in most previous studies on personality types. We therefore now added a paragraph in ll. 81-92 explicitly stating the aim of the study and the purpose of the literature review, structured the introduction in three parts (general, review part, research gap and novelty part) to guide the reader through this part of the study and adjusted the study title.*

7) from row 227 to row 229: I would extend this concept

*Response: We expanded the paragraph so the purpose of the inclusion of these external measures becomes more clear.*

8) from row 233 to row 244: I would insert a table representing the elements described

*Response: Thanks very much for this suggestion, we now have a table	included in ll.310 hopefully improving the understanding of the sample composition for the reader.*

9) from row 291 to row 321and from row 325 to row 353: The meaning of these methods should be clarified in relation to the type of data examined. this will allow a perfect understanding of the results even for non-expert colleagues in data analysiss

*Response: Thanks again for this suggestion, we now have added several sentences in ll.380-391 explaining the methods with respect to personality types and think this really improves the understanding also for non-expert colleagues.*

10) from row 354 to row 357: I would delete this sentence, there are recent studies that question this claim (see Matthijs J Warrens On the Equivalence of Cohen’s Kappa and the Hubert-Arabie Adjusted Rand Index February 2008 Journal of Classification 25 (2): 177-183)

*Response: Thanks for pointing out the literature, we now have rephrased this part of the manuscript in ll.444-446.*

11) from row 358 to row 383: These are basic concepts for which the paragraph can be reduced in size

*Response: We agree that these concepts are basic, but without this paragraph, the experienced reader would not now why we chose the specific internal validity criteria we chose from the huge universe of internal validity criteria formulas. We now have added a sentence in ll. 448 - 449 explaining the purpose of this paragraph and a sentence in ll. 469 470 explaining the choice of internal validity criterias with respect to personality type data.*

12) from row 384 to row 435: Insert a figure that graphically describes the procedure

*Response: Thanks for this suggestion, we now have added a figure describing the procedure and think that this improves the understandability of the complicated methods in this study.*

13) from row 677 to row 680: delete this sentence

*Response: As the link and connection to previous evidence is an important gap in previous papers on personality types, we would like to keep this sentence in the discussion.*
