# Supplementary material for: Personality types revisited–a literature-informed and data-driven approach to an integration of prototypical and dimensional constructs of personality description
Source: PLoS One. 2021 Jan 7;16(1):e0244849. doi: 10.1371/journal.pone.0244849 (PMC7790254; doi:10.1371/journal.pone.0244849)
Supplement: Attachment — Submitted filename: Response to the Reviewers.docx [file pone.0244849.s002.docx]

In the following, all line numbers refer to the version of the manuscript with tracked changes.

Reviewer 1:

1) The tables have no definitions in the legend for the different terms and abbreivations - I am not entirely aware of the author guidelines for this journal, but I think it is much needed.

*Response: Thanks to this suggestion we have reviewed all our tables for abbreviations that are not explained and included them in the respective notes.*

2) The authors rigthly included a reference to the now approved ICD-11 PD classification (line 825). However, the authors should provide the correct reference:

*Response: We have rephrased ll. 838-841 to also include a reference to the ICD-11 PD model.*
